# Spontaneous first impressions emerge from brief training

**DOI:** 10.1038/s41598-021-94670-y

**Published:** 2021-07-22

**Authors:** Ruth Lee, Jonathan C. Flavell, Steven P. Tipper, Richard Cook, Harriet Over

**Affiliations:** 1 University of York, Heslington, York, YO10 5DD UK; 2 Birkbeck, University of London, Malet Street, London, WC1E 7HX UK; 3 Present Address: School of Psychology, Queen’s University Belfast, David Keir Building, 18-30 Malone Road, Belfast, BT9 5BN UK

**Keywords:** Psychology, Human behaviour

## Abstract

People have a strong and reliable tendency to infer the character traits of strangers based solely on facial appearance. In five highly powered and pre-registered experiments, we investigate the relative merits of learning and nativist accounts of the origins of these first impressions. First, we test whether brief periods of training can establish consistent first impressions de novo. Using a novel paradigm with Greebles—a class of synthetic object with inter-exemplar variation that approximates that seen between individual faces—we show that participants quickly learn to associate appearance cues with trustworthiness (Experiments 1 and 2). In a further experiment, we show that participants easily learn a two-dimensional structure in which individuals are presented as simultaneously varying in both trustworthiness and competence (Experiment 3). Crucially, in the final two experiments (Experiments 4 and 5) we show that, once learned, these first impressions occur following very brief exposure (100 ms). These results demonstrate that first impressions can be rapidly learned and, once learned, take on features previously thought to hold only for innate first impressions (rapid availability). Taken together, these results highlight the plausibility of learning accounts of first impressions.

## Introduction

Upon meeting a stranger, observers quickly draw inferences about their apparent trustworthiness, honesty, competence and intelligence^1–3^. These first impressions appear to exert a powerful influence over behaviour and can result in systematic bias^4–6^. The behavioural consequences of these trait inferences are particularly troubling because while some first impressions appear to be veridical^7–9^, many others bear little or no resemblance to the actual character traits of the individuals being judged^10,11^.

The inferences that we make about traits from faces are often argued to be partly a product of innate cognitive architecture specialized for first impressions. According to this theoretical position, distinguishing friends from foe and leaders from followers was so crucial to the survival of our species that we evolved mechanisms for making spontaneous judgments from others’ appearance^12–15^.

According to an influential alternative framework, Trait Inference Mapping (TIM), first impressions are largely products of learned associations between points in face space and trait space^16,17^ These mappings allow excitation to spread automatically from perceptual descriptions of face shape to representations of particular trait profiles. Exposure to consistent depictions of “good guys” and “bad guys”, “leaders” and “followers” in illustrated storybooks, film, television, ritual, art, and iconography^18–21^ may lead different individuals within a society to acquire similar face–trait mappings—so-called consensus impressions. Other, more ‘idiosyncratic’ mappings may be acquired as a result of direct social interactions with others^12,22–26^.

Proponents of both theoretical positions agree that at least some first impressions are learned^12,16,27–34^. Evidence in favour of this claim comes from data showing that participants form first impressions from cultural cues. For example, children and adults from Western cultures typically judge individuals who wear glasses to be more intelligent than individuals who do not wear glasses^35^. As glasses are an invention of relatively recent human history, these first impressions cannot be the result of gene-based natural selection^16^. Consistent with at least some role for learning, other research has shown that there appear to be systematic cultural differences in first impressions from appearance^17^ and that it is possible to modify pre-existing first impressions of faces with training^28,36^. More recently, a twin study confirmed that individual differences in first impressions are driven mostly by the environment^12^.

Nativist accounts hold that where first impressions are innate they can be recognized by three features. Innate first impressions (1) emerge early in development, (2) show broad cross-cultural similarity and (3) are accessible following very rapid presentation of stimuli^12–15,22,37^. In relation to the latter, evidence that observers form consistent first impressions even when faces are presented for as little as 100 ms has been taken as evidence that they are likely to be innately specified^3,13^.

According to TIM, on the other hand, mappings between face space and trait space are the products of learning^16^. TIM predicts that adults will be able to learn first impressions from relatively brief experience. Rather than requiring protracted social experience over several years, learning and generalisation may occur with brief training. Furthermore, once learned, these first impressions will become rapidly available. That is, first impressions acquired through learning will quickly take on qualities previously assumed to apply specifically to mappings based on innate architecture.

The study of appearance-trait learning in the lab is not straightforward. One approach is to attempt to modify existing patterns of face-trait mappings by providing participants with novel face-trait experience^23,28,29^. For example, having learned that a particular individual is untrustworthy or trustworthy, participants are less likely to trust people who resemble that person in future interactions^28,36^. However, the learning required to modify a mature set of mappings and that required to establish mappings de novo may differ in important respects. Participants arrive with firm preconceptions about the relationship between facial appearance and traits^37,38^. Lab-based face-trait experience that violates those expectations may be surprising and thereby exert a disproportionate influence on learning^39,40^. There is also some indication that new learning that contradicts a previously-learned rule tends to manifest only in specific contexts^41,42^. Most importantly in this context, it is not possible to use this type of paradigm to determine whether first impressions acquired purely through learning can become rapidly available.

In the present paper, we seek to develop a second, complementary approach to the study of appearance-trait learning, whereby a novel stimulus category is used as a proxy for faces. By presenting participants with novel stimuli, with which they have had no previous experience, researchers can examine how trait inferences emerge de novo as a function of correlated appearance-trait experience. We illustrate this approach using Greebles^43,44^. Greebles are a class of synthetic object developed to study the emergence of perceptual expertise. Every Greeble has a vertical central part and four protruding parts. The variation between Greeble exemplars broadly replicates that seen between individual faces. Thus, Greebles can be categorised into two ‘genders’ (glips and ploks, defined by the orientation of the protruding parts: upward or downward,) and five ‘families’, (Samar, Osmit, Galli, Radok, and Tasio, defined by the shape of the central part). The presence of this inter-exemplar structure makes them an ideal proxy for faces in studies of appearance-trait learning.

In five experiments, we examine whether participants can learn that some Greebles are more trustworthy and competent than others. These attributes were chosen because research suggests that trait perception is structured along these two dimensions ^45,46^. In all experiments, we measure whether adult participants exhibit learning about individual Greebles that generalises to novel Greebles of similar appearance. In the crucial fourth and fifth experiments, we measure whether first impressions acquired through learning take on qualities previously assumed to apply specifically to mappings based on innate architecture. That is, we measure whether first impressions acquired through training occur rapidly (following 100 ms presentation).

Informed consent was obtained from all participants. All experiments were carried out in accordance with the Declaration of Helsinki. Ethical approval for all experiments was received from the research ethics committee of the Department of Psychology, University of York, protocol number 820. Data for all experiments are available in the OSF repository at https://osf.io/ub6th/?view_only=72831b53995543659182f6db6f254231^47^.

## Experiment 1

### Method

#### Experimental overview

Participants first completed a training procedure in which they encountered Greebles from two families. The Greebles from one family acted in a way that was consistently trustworthy. The Greebles from the second family acted in a way that was consistently untrustworthy. In a subsequent test, we measured whether participants learned about the apparent trustworthiness of the Greebles from this procedure, and whether they generalized any learning to novel but similar looking Greebles from the same family. Our preregistered inclusion criteria and analysis plans for this and all subsequent experiments are available at https://osf.io/ub6th/?view_only=72831b53995543659182f6db6f254231^47^. This experiment, and all subsequent experiments save for Experiments 4 and 5, were presented using Qualtrics software^48^.

#### Participants

Forty participants (*M*_age_ = 32.93 years, *SD*_age_ = 11.19 years, range: 18–60 years, 11 males) were recruited through www.prolific.co. All participants in this and all subsequent experiments indicated that they were primarily resident in the UK. Participants each received a small honorarium for taking part. In order to participate, participants had to be aged 18 years old or more, speak English as a native language, and reside in the UK at the time of testing. We planned to exclude participants whose task completion time was more than three standard deviations below the mean. However, no participants met this criterion. No participants in the current study completed more than one experiment.

Sample size was determined a priori by a power analysis conducted using MorePower 6.0.4^49^. Power and alpha were set at the conventional levels of 0.8 and 0.05 respectively. Pilot data yielded a large effect size (partial eta-squared of 0.38) for the effects of interest. The analysis indicated that a sample size of 40 ensured adequate power for a 2 × 2 repeated measures ANOVA.

#### Contextualization

The experiment began with a brief introduction designed to demonstrate that Greebles are intentional agents, and thus could plausibly participate in the kinds of actions that participants would hear about during the training trials. Four pictures were individually presented, each showing a different Greeble on a background portraying an outdoor scene. Each picture was accompanied by a text describing an action that demonstrated neither trustworthiness nor competence (e.g., ‘Here is a Greeble going for a walk’). The Greebles presented during the introduction were from different families to those used in the experiment. Note that at no stage during this or subsequent experiments were participants informed that Greebles can be categorized by family or gender.

#### Training procedure

**Figure 1:**
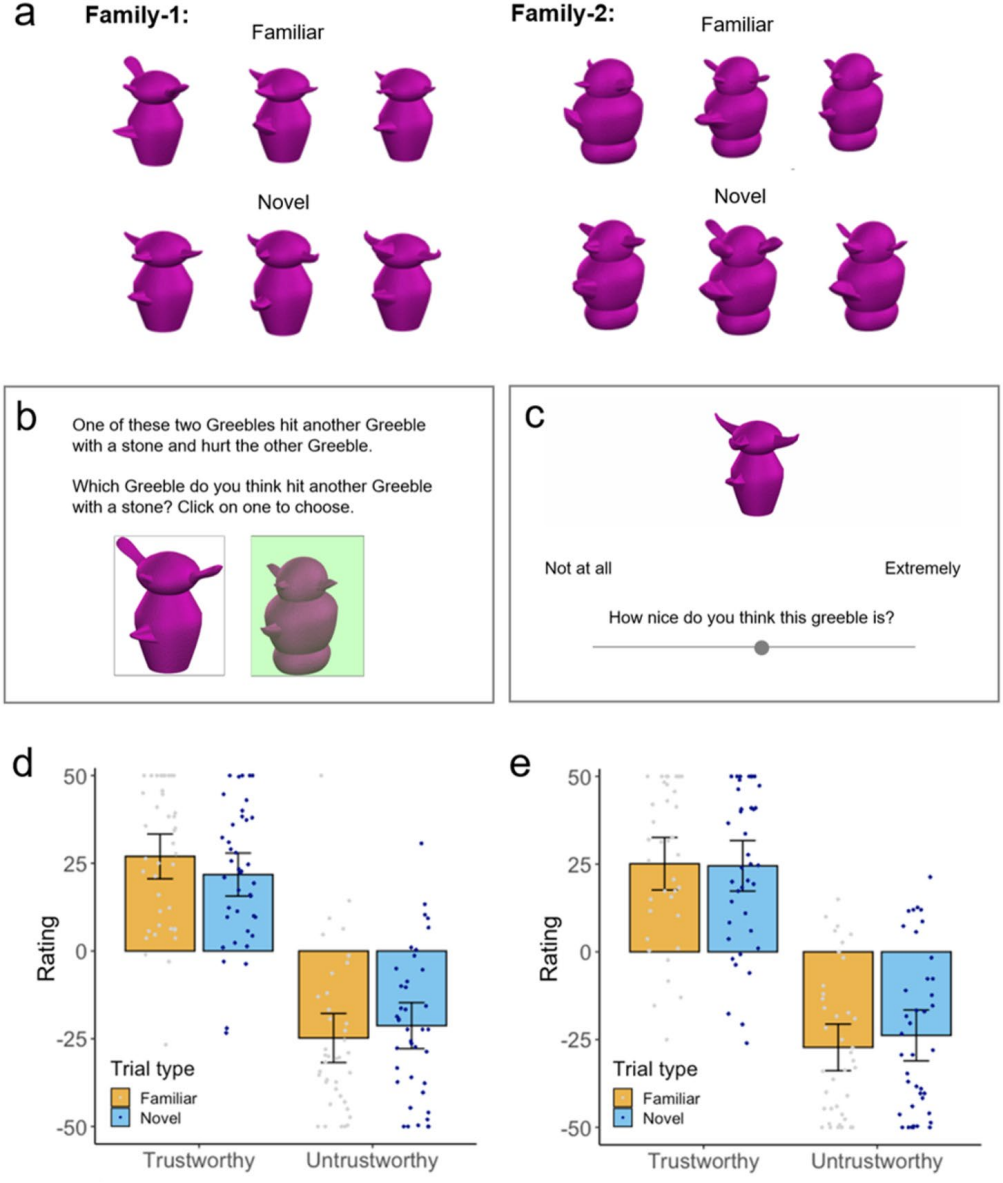
(**a**) The familiar and novel Greebles used in Experiments 1, 2, and 4. (**b**) Example display from a training trial. (**c**) Example display from a test trial. (**d**) Results from Experiment 1. (**e**) Results from Experiment 2. Error bars represent 95% confidence intervals around the mean. Stimulus images courtesy of Michael J. Tarr, Carnegie Mellon University, http://www.tarrlab.org/.

Participants completed 72 training trials. Each training trial depicted a male Greeble from Family-1 and a male Greeble from Family-2 (Fig. 1a). The two Greebles were presented side-by-side below a text description of an action and its consequence (Fig. 1b).

On half (36) of the trials, actions were positively valenced on the dimension of trustworthiness. The remaining 36 trials presented actions that were negatively valenced on the dimension of trustworthiness. Trustworthy actions involved sharing behaviors (e.g., ‘One of these two Greebles shared some nuts with another Greeble so that the other Greeble wouldn’t be hungry’), helping behaviors (e.g., ‘One of these two Greebles went up a ladder for another Greeble because the other Greeble was scared of heights’), and caring behaviors (e.g., ‘One of these two Greebles read a story to another Greeble and the other Greeble really enjoyed it’).

The remaining 36 trials presented actions that were negatively valenced on the dimension of trustworthiness. Untrustworthy actions involved either refusing to engage in helping, sharing, or caring behavior (e.g., ‘One of these two Greebles refused to share a shelter with another Greeble and let the other Greeble get wet’), or behaviors that were antithetical to sharing, helping, or caring (e.g., ‘One of these two Greebles threw eggs at another Greeble and made the other Greeble upset’).

On each trial, participants were asked to choose which Greeble they thought had performed the action. When participants clicked on their chosen Greeble, the background to that picture was illuminated (Fig. 1b). If participants chose correctly, then they saw a further picture informing them that their choice was correct (‘Yes! This Greeble [past action description]’. If they chose incorrectly, they saw the second picture (‘No! This Greeble [past action description]’). Regardless of whether a participant answered correctly or incorrectly, a green tick was displayed over the correct Greeble and a red cross over the incorrect Greeble.

For half of participants, Family-1 was associated with trustworthy actions and Family-2 was associated with untrustworthy actions. For the other half of participants, this mapping was reversed. Events were presented in one of two random orders. Half of participants viewed events in one order, and half in the other. For each type of behavior (sharing, helping, and caring), the relative position of the Greebles from Family-1 and Family-2 was counterbalanced.

#### Test procedure

Following training, participants completed a test procedure (Fig. 1c). Each of the 12 test trials presented a single Greeble centrally. Participants rated how nice the Greeble was on a sliding scale ranging from ‘Not at all’ (representing − 50) to ‘Extremely’ (representing + 50). ‘Nice’ was defined for participants before they began the test trials: ‘*Nice* means socially warm and pleasant.’

Participants rated the six Greebles on which they had been trained (henceforth ‘familiar’ Greebles) and six Greebles (3 from each trained family) that they had not yet encountered (henceforth ‘novel’ Greebles). The 12 test trials were presented in a different random order for each participant. For each participant, we computed four average ratings: trustworthy family (familiar Greebles), trustworthy family (novel Greebles), untrustworthy family (familiar Greebles), untrustworthy family (novel Greebles).

### Results

The average trust ratings (Fig. 1d) were analysed using ANOVA with Trustworthiness (trustworthy, untrustworthy Greebles) and Trial Type (familiar, novel) as within-subjects factors. The analysis revealed a main effect of Trustworthiness [*F*(1,39) = 89.45, *p* < .001, η^2^ = 0.70] indicating that participants learned about the relative trustworthiness of the Greebles from the training. The analysis yielded no effect of Trial Type [*F*(1,39) = 0.27, *p* = .630, η^2^ = 0.01]. There was a marginal interaction between trustworthiness and Trial Type [*F*(1,39) = 4.08, *p* = .050, η^2^ = 0.10]. However, ratings for familiar and novel Greebles did not differ significantly for trustworthy [*t*(39) = 1.81, *p* = .077, *d* = 0.29] or untrustworthy Greebles [*t*(39) = 1.33, *p* = .191, *d* = 0.21]. These results indicate that participants quickly learnt about the character traits of Greebles. This learning generalised to novel Greebles with little or no decrement.

In further exploratory analyses, we sought to ensure that there was a significant difference for both the familiar and novel trials when considered independently. In order to assess this, we ran two paired samples t-tests. These tests demonstrated that there was a significant difference between trustworthy and untrustworthy Greebles both when they were familiar to participants (*t*(39) = 9.15, *p* < .001, *d* = 1.45) and when they were novel (*t*(39) = 8.21, *p* < .001, *d* = 1.30).

## Experiment 2

### Method

#### Experimental overview

In our first experiment, we found that participants were able to learn a relationship between Greeble appearance cues and a character trait (trustworthiness). In Experiment 2, we sought to determine whether this mapping can be acquired with even less experience, using half the number of training trials.

#### Participants

Forty participants were recruited through www.prolific.co (*M*_age_ = 35.38 years, *SD*_age_ = 10.76 years, range: 20–68 years, 12 males). The inclusion criteria were the same as for Experiment 1. No participants were removed from the analyses. Participants each received a small honorarium.

#### Training and test procedure

The design, materials, procedure and data scoring were identical to those used in Experiment 1. The only difference was that there were 36 rather than 72 training trials.

### Results

The average trust ratings (Fig. 1e) were analysed using ANOVA with Trustworthiness (trustworthy, untrustworthy Greebles) and Trial Type (familiar, novel) as within-subjects factors. The analysis revealed a main effect of Trustworthiness [*F*(1,39) = 62.77, *p* < .001, η^2^ = 0.62] indicating that participants learned about the relative trustworthiness of the Greebles from the training. However, the analysis yielded no effect of Trial Type [*F*(1,39) = 0.98, *p* = .328, η^2^ = 0.03] and no Trustworthiness × Trial Type interaction [*F*(1,39) = 1.33, *p* = .257, η^2^ = 0.03]. Despite the abbreviated training procedure, participants learned the appearance-trustworthiness mapping and applied it to novel exemplars without decrement.

In further exploratory analyses, we sought to ensure that there was a significant difference for both the familiar and novel trials when considered independently. Two paired-samples t-tests demonstrated that there was a significant difference between trustworthy and untrustworthy Greebles both when they were familiar to participants (*t*(39) = 7.93, *p* < .001, *d* = 1.25) and when they were novel (*t*(39) = 7.36, *p* < .001, *d* = 1.16).

## Experiment 3

### Method

#### Experimental overview

In our next experiment, we examined whether we could replicate these findings when participants were asked to learn more complex patterns of appearance-trait mappings. We took advantage of the fact that Greebles have both families and genders. Importantly, different appearance cues define family membership (shape of central part) and gender membership (orientation of protruding parts). For example, a male Greeble from Family-1 might closely resemble another male Greeble from Family-1, and bear little resemblance to a female Greeble from Family-2. However, a male Greeble from Family-1 would share some appearance cues with a female Greeble from Family-1 and share other appearance cues with a male Greeble from Family-2.

We examined whether participants could learn that one set of appearance cues (e.g., gender features) were predictive of competence, and another set of cues (e.g., family features) were predictive of trustworthiness. The structure of this task more closely mirrors the nature of appearance-trait covariation in the real world^46^: prominent models of social perception argue that individuals can vary independently in trustworthiness and competence^50^.

#### Participants

One hundred and twenty-eight participants were recruited through www.prolific.co (*M*_age_ = 37.40 years, *SD*_age_ = 14.1 years, range: 18–82 years, 49 males). Our preregistered data collection and analysis plan specified 126 participants, based on power analysis. However, to achieve an equal number of participants in each counterbalancing condition we required 128 participants. We therefore collected an additional two participants. The inclusion criteria were the same as for Experiment 1. No participants were removed from the analyses. Participants each received a small honorarium. Power analysis conducted a priori with MorePower 6.0.4^49^ with power and alpha at the conventional levels of 0.8 and 0.05 respectively indicated that a sample of 126 was needed to provide power of 0.8 for a for a 2 × 2 repeated measures ANOVA, assuming an effect size of 0.06.

#### Training procedure

**Figure 2:**
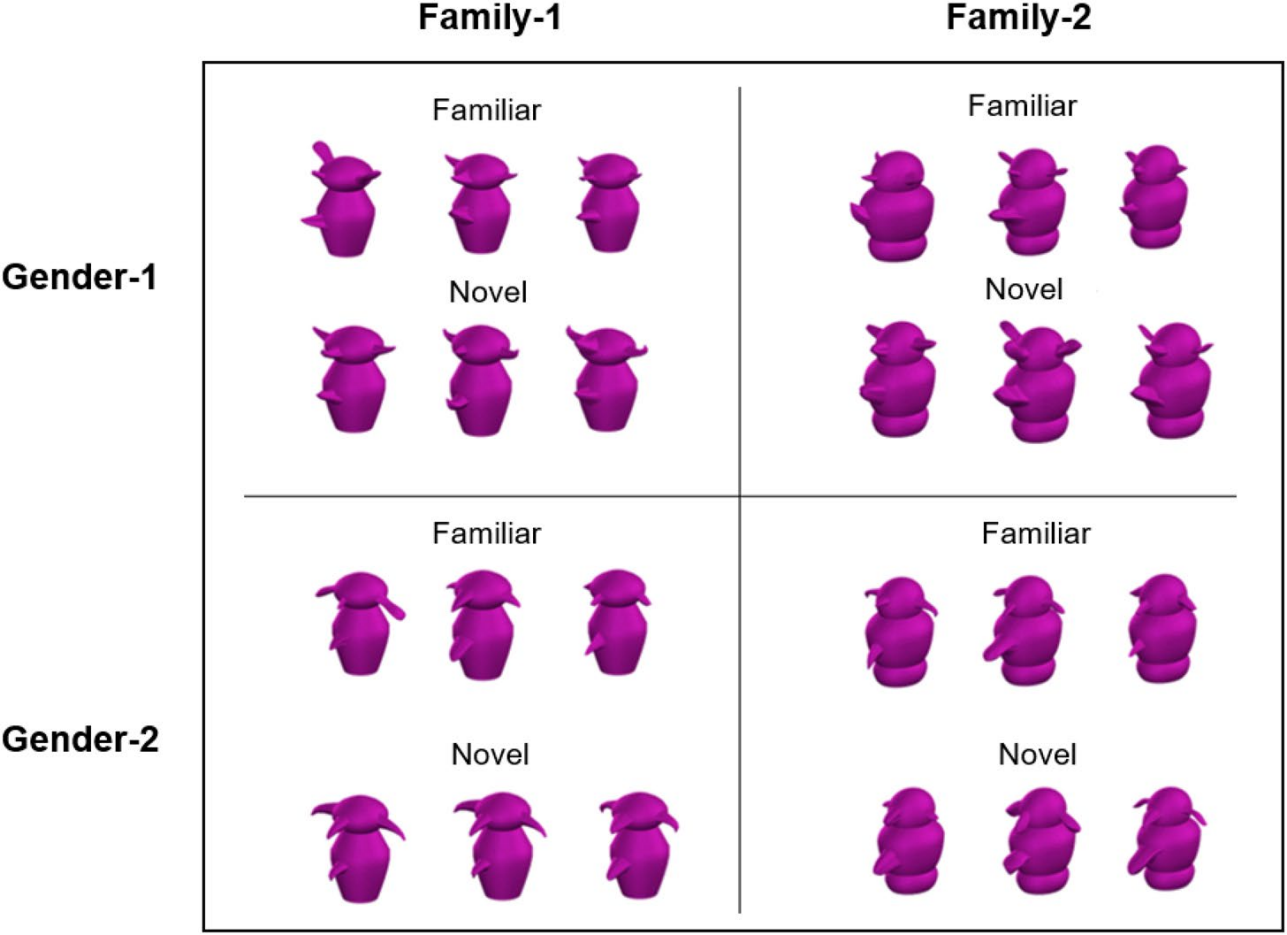
The 24 Greebles used in Experiments 3 and 5 included exemplars from two genders and two families. Stimulus images courtesy of Michael J. Tarr, Carnegie Mellon University, http://www.tarrlab.org/.

Participants completed 72 training trials, during which they encountered 12 Greebles (Fig. 2). Three were males from Family-1, three were females from Family-1, three were males from Family-2, and three were females from Family-2. As before, each training trial presented two Greebles side-by-side below an event description. In half (36) of these events the protagonists engaged in behaviours demonstrating high or low trustworthiness, and in the other 36 events they demonstrated high or low competence.

The events described during trustworthiness training trials were identical to those used in Experiment 2. During competence training trials, competent actions demonstrated intelligence (e.g., ‘One of these two Greebles got some difficult sums right and worked out how much money was left for the week’), innovation (e.g., ‘One of these two Greebles designed a new kind of aeroplane that could fly further than ever before’), and knowledge (e.g., ‘One of these two Greebles learned all about rare birds and then spotted one in the woods’). Incompetent actions involved behaviors demonstrating lack of intelligence (e.g., ‘One of these two Greebles read a map upside down and got lost’), failures of innovation, (e.g., ‘One of these two Greebles made mistakes in building a rocket and it crashed into a tree’), or lack of knowledge (e.g., ‘One of these two Greebles couldn’t learn to ride a canoe and fell into the river’).

Participants were randomly allocated to one of eight counterbalancing conditions (Table 1). For 50% of participants, gender cues predicted the trustworthiness of the Greebles and family cues predicted their competence. For the remaining participants, family cues predicted the trustworthiness of the Greebles and gender cues predicted their competence. Events were presented in one of two random orders. Since each Greeble belonged both to a particular family and to a particular gender, Greebles were presented as varying in both trustworthiness and competence. Within each set of 36 trustworthiness-related events and each set of 36 competence-related events, each of the 12 individual target Greebles was presented three times to each participant. The second Greeble was always matched to the target Greeble on the valence of the trait irrelevant to the event. That is, where an event concerned trustworthiness, either both Greebles presented as competent, or both as incompetent.

**Table 1:** The eight appearance-trait mappings trained in Experiment 3.

	Counterbalancing condition							
	1	2	3	4	5	6	7	8
Gender-m	C+	C−	C−	C+	T+	T−	T+	T−
Gender-f	C−	C+	C+	C−	T−	T+	T−	T+
Family-1	T+	T−	T+	T−	C+	C−	C−	C+
Family-2	T−	T+	T−	T+	C−	C+	C+	C−

C+ and C− denote high and low competence.

T+ and T− denote high and low trustworthiness.

#### Test procedure

Each test trial presented a single Greeble. Participants were asked to rate the Greeble shown for both trustworthiness and competence using a sliding scale. The order of presentation of the trustworthiness scale and competence scale was counterbalanced, such that half of participants saw the trustworthiness question above the competence question, and half saw the competence question above the trustworthiness question. Twelve test trials presented the familiar Greebles that had been encountered during training. Twelve test trials presented novel Greebles that had not been seen before (Fig. 2). Three were males from Family-1, three were females from Family-1, three were males from Family-2, and three were females from Family-2. The 24 test trials were presented in a different random order for each participant.

### Results

For each participant, we computed eight average trust and eight average competence ratings from the three factors of: trustworthiness (high/low) × competence (high/low) × type (novel/familiar). Following our pre-registered plan, two repeated measures Analyses of Variance (ANOVA) were conducted separately on the dependent variables of trustworthiness and competence ratings. In each ANOVA, Trustworthiness (trustworthy, untrustworthy), Competence (competent, incompetent), and Trial Type (familiar, novel Greebles) were within-subjects factors.

**Figure 3:**
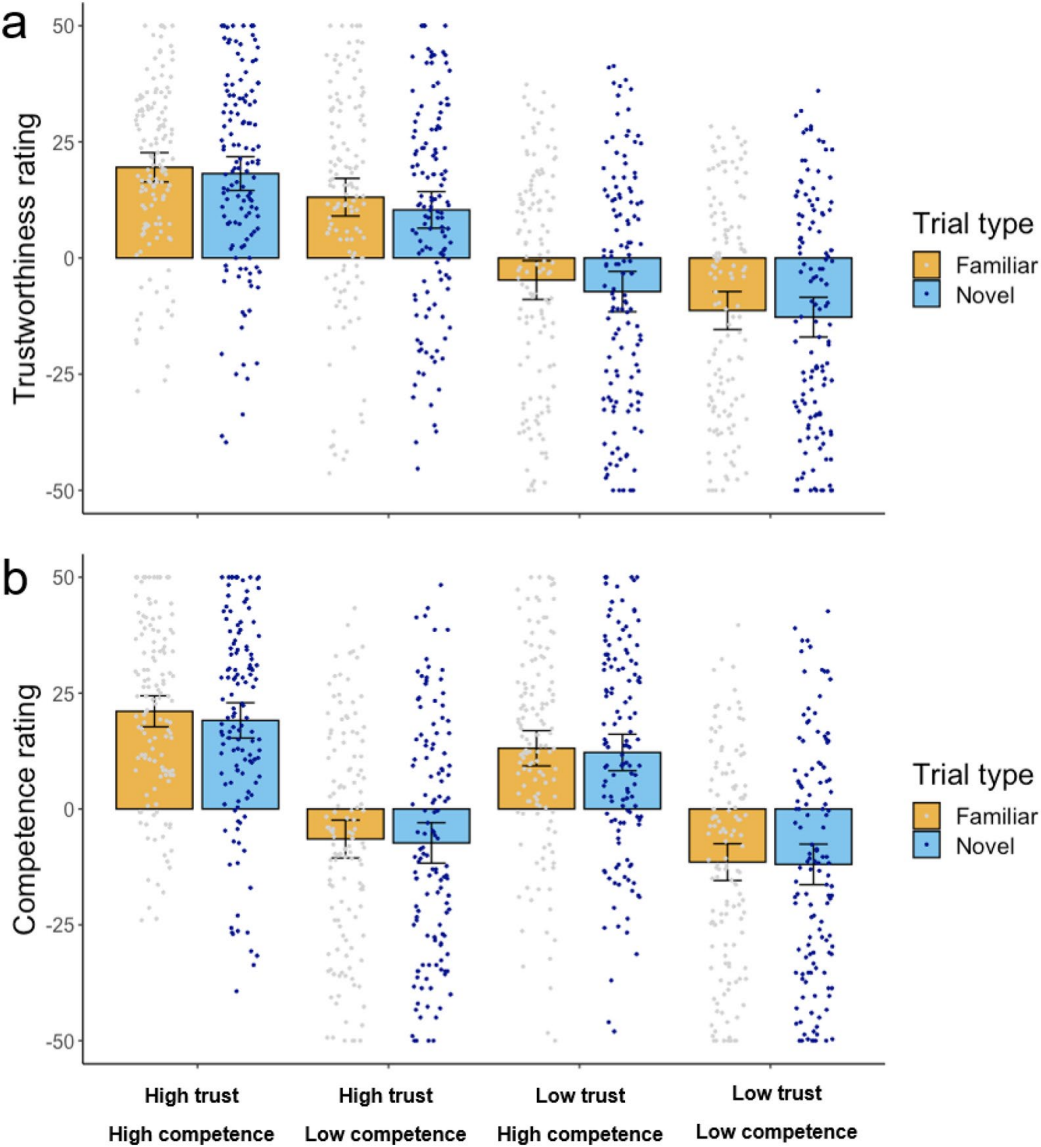
(**a**) Results from Experiment 3, judgements of trustworthiness. (**b**) Results from Experiment 3, judgements of competence. Error bars represent 95% confidence intervals around the mean.

Trustworthiness ratings (Fig. 3a): The analysis revealed a main effect of Trustworthiness, such that trustworthy Greebles were rated as more trustworthy than untrustworthy Greebles [*F*(1,127) = 68.48, *p* < .001, η^2^ = 0.35] and a main effect of competence, such that competent Greebles were also rated as more trustworthy than incompetent Greebles [*F*(1,127) = 13.15, *p* < .001, η^2^ = 0.09]. There was also a main effect of Trial Type [*F*(1,127) = 11.58, *p* < .001, η^2^ = 0.08], such that familiar Greebles were rated as more trustworthy than novel Greebles. No significant interactions were observed (all *F*’s < 0.1, all *p*’s > .32).

We further ran exploratory paired samples t-tests in order to ensure that when familiar and novel trials were considered independently, significant differences remained in trust ratings. There was a significant difference in trust ratings between trustworthy and untrustworthy Greebles both when they were familiar to participants (*t*(127) = 8.31, *p* < .001, *d* = 0.73) and when they were novel (*t*(127) = 7.91, *p* < .001, *d* = 0.70). There was also a significant difference in trust ratings between competent and incompetent Greebles that were familiar (*t*(127) = 3.36, *p* = .001, *d* = 0.30) and those that were novel (*t*(127) = 3.35, *p* = .001, *d* = 0.30) to participants.

Competence ratings (Fig. 3b): The analysis revealed a main effect of Trustworthiness, such that trustworthy Greebles were rated as more competent than untrustworthy Greebles [*F*(1,127) = 8.62, *p* < .005, η^2^ = 0.06] and a main effect of competence, such that competent Greebles were also rated as more competent than incompetent Greebles [*F*(1,127) = 89.52, *p* < .001, η^2^ = 0.41]. There was no main effect of Trial Type [*F*(1,127) = 1.97, *p* = .152, η^2^ = 0.02]. No significant interactions were observed (all *F*’s < 2.67, all *p*’s > .105).

We again ran paired samples t-tests in order to ensure that when familiar and novel trials were considered independently, significant differences remained in competence ratings. There was a significant difference in competence ratings between competent and incompetent Greebles both when they were familiar to participants (*t*(127) = 9.65, *p* < .001, *d* = 0.85) and when they were novel (*t*(127) = 8.57, *p* < .001, *d* = 0.76). There was also a significant difference in competence ratings between trustworthy and untrustworthy Greebles that were familiar to participants (*t*(127) = 3.04, *p* = .003, *d* = 0.27) and those that were novel (*t*(127) = 2.54, *p* = .012, *d* = 0.22).

Experiment 3 demonstrates that people are able to quickly learn more complex appearance-trait mappings, and that they generalize this learning to novel individuals who resemble trained exemplars.

## Experiment 4

### Method

#### Experimental overview

In our next experiment, we examine whether first impressions acquired through learning become available even after rapid presentation. Nativist accounts have previously gained support from evidence suggesting that observers form consistent first impressions of apparent trustworthiness even when faces are presented for as little as 100 milliseconds^3,13,51,52^. In this experiment, we test whether evidence for rapid availability is equally compatible with a learning-based account. We measure whether learned associations lead to rapid first impressions by restricting viewing time at test to 100 ms. The experiment was presented using Gorilla software (gorilla.sc)^53,54^.

#### Participants

Forty participants were recruited through www.prolific.co (*M*_age_ = 33.9 years, *SD*_age_ = 12.12 years, range: 19–59 years, 16 males). The inclusion criteria were the same as for Experiment 1. No participants were removed from the analyses. Participants each received a small honorarium.

#### Training and test procedure

Design, materials, and procedure were identical to those used in Experiment 2, save that test trials were presented for only 100 ms rather than self-paced. Following previous research on first impressions and in other areas of cognitive psychology, we employed a binocular noise mask^35,55,56^.

**Figure 4:**
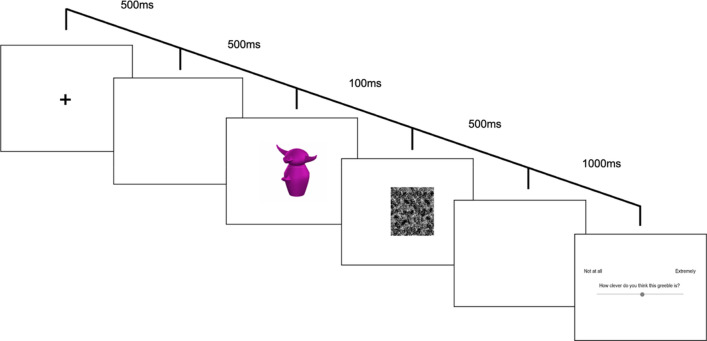
Trial structure in Experiment 4. Stimulus images courtesy of Michael J. Tarr, Carnegie Mellon University, http://www.tarrlab.org/.

Participants were told that they would only see each Greeble for a very brief moment at test. At the outset of each test trial, participants viewed a fixation cross at the centre of the screen for 500 ms followed by 500 ms of blank screen. A Greeble then appeared for 100 ms and was then covered by a noise mask for 500 ms (Fig. 4), followed by a blank screen for 1000 ms. Participants then gave their ratings. The square mask had sides of ~ 147 mm.

### Results

**Figure 5:**
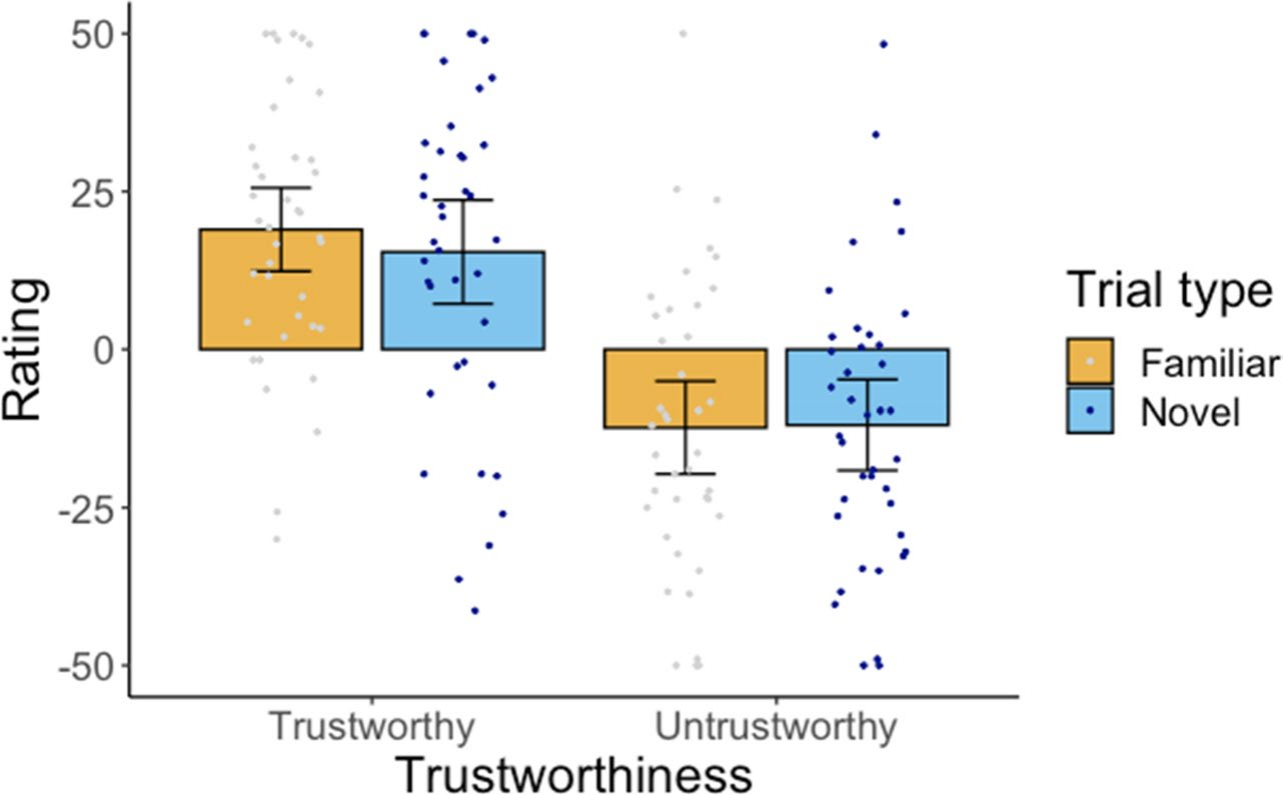
Results from Experiment 4. Error bars represent 95% confidence intervals around the mean.

The average trustworthiness ratings (Fig. 5) were analysed using ANOVA with Trustworthiness (trustworthy, untrustworthy) and Trial Type (familiar, novel) as within-subjects factors. The analysis revealed a main effect of Trustworthiness [*F*(1,39) = 29.87, *p* < .001 ., η^2^ = 0.43] indicating that participants learned about the relative trustworthiness of the Greebles from the training. The analysis yielded no effect of Trial Type [*F*(1,39) = 0.66, *p* = .423, η^2^ = 0.02] and no Trustworthiness × Trial Type interaction [*F*(1,39) = 0.63, *p* = .434, η^2^ = 0.02]. Thus, participants formed first impressions of the Greebles presented at test regardless of whether exemplars were familiar or novel. These first impressions emerged even though presentation time was extremely brief (100 ms).

In further exploratory analyses, we ran two paired samples t-tests in order to ensure that there was a significant difference for both the familiar and novel trials when considered independently. There was a significant difference between trustworthy and untrustworthy Greebles both when they were familiar to participants (*t*(39) = 5.87, *p* < .001, *d* = 0.93) and when they were novel (*t*(39) = 4.24, *p* < .001, *d* = 0.67).

## Experiment 5

### Method

#### Experimental overview

In our final experiment, we further test whether evidence for rapid availability is compatible with a learning-based account. As in Experiment 4, we measure whether learned associations lead to rapidly available first impressions by restricting viewing time at test to 100 ms. In this experiment, however, we ask participants to learn a more complex two-dimensional structure in which Greebles vary in both warmth and competence. The experimental design is thus extremely similar to Experiment 3 with the exception that viewing time at test is restricted to 100 ms.

#### Participants

128 participants were recruited through www.prolific.co (*M*_age_ = 34.71, *SD*_age_ = 12.85 years, range: 18–64 years, 45 males; three participants preferred to self-describe their gender). The inclusion criteria were the same as for Experiment 1. No participants were removed from the analyses. Participants each received a small honorarium.

#### Training and test procedure

Design, materials, and procedure were identical to those used in Experiment 3, save for three differences. First, test trials were presented for only 100 ms. Second, to facilitate this rapid presentation, the experiment was presented using the Gorilla Experiment Builder (www.gorilla.sc)^53,54^. Third, events during the training phase were presented in a different random order for each participant rather than being presented in one of two random orders.

### Results

**Figure 6:**
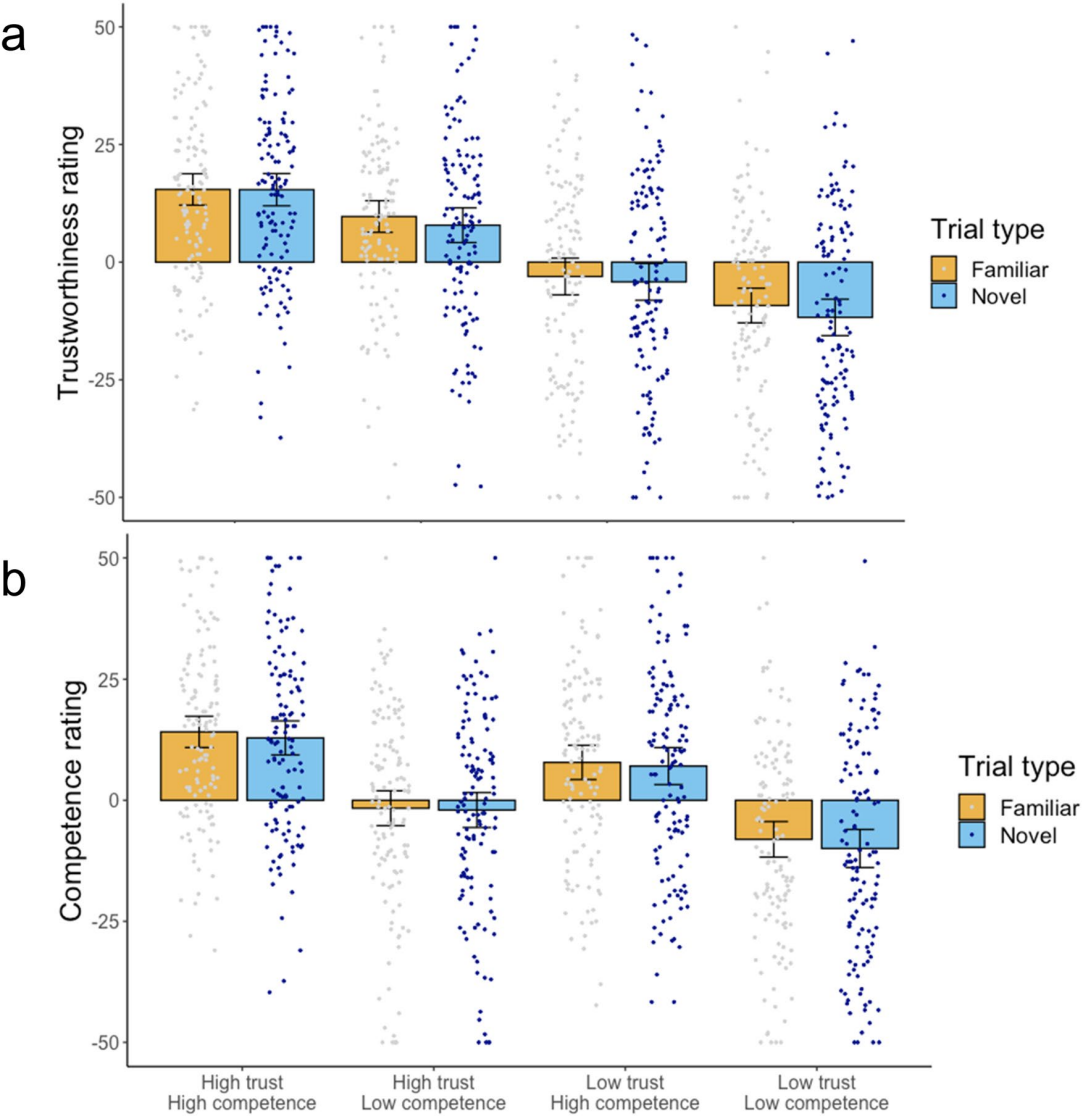
(**a**) Results from Experiment 5, judgements of trustworthiness. (**b**) Results from Experiment 5, judgements of competence. Error bars represent 95% confidence intervals around the mean.

As in Experiment 3, we computed eight average trust and eight average competence ratings from the three factors of: trustworthiness (high/low) × competence (high/low) × type (novel/familiar) for each participant. Following our pre-registered plan, we conducted two repeated measures Analyses of Variance (ANOVA) separately on the dependent variables of trustworthiness ratings (Fig. 6a) and competence ratings (Fig. 6b). In each ANOVA, Trustworthiness (trustworthy, untrustworthy), Competence (competent, incompetent), and Trial Type (familiar, novel Greebles) were within-subjects factors.

Trustworthiness ratings (Fig. 3a): The analysis revealed a main effect of trustworthiness, such that trustworthy Greebles were rated as more trustworthy than untrustworthy Greebles [*F*(1,127) = 54.50, *p* < .001, η^2^ = 0.32] and a main effect of competence, such that competent Greebles were also rated as more trustworthy than incompetent Greebles [*F*(1,127) = 14.72, *p* < .001, η^2^ = 0.10]. There was also a main effect of Trial Type [*F*(1,127) = 4.49, *p* = .036, η^2^ = 0.03], such that familiar Greebles were rated as more trustworthy than novel Greebles. No significant interactions were observed (all *F*’s < 1.4, all *p*’s > .244).

We further ran paired samples t-tests in order to ensure that when familiar and novel trials were considered independently, significant differences remained in warmth ratings. There was a significant difference in trust ratings between trustworthy and untrustworthy Greebles both when they were familiar to participants (*t*(127) = 7.39, *p* < .001, *d* = 0.65) and when they were novel (*t*(127) = 7.40, *p* < .001,* d* = 0.66). There was also a significant difference in trust ratings between competent and incompetent Greebles that were familiar (*t*(127) = 3.25, *p* = .001,* d* = 0.29) and novel (*t*(127) = 3.90, *p* < .001,* d* = 0.34) to participants.

Competence ratings (Fig. 3b): The analysis revealed a main effect of trustworthiness, such that trustworthy Greebles were rated as more competent than untrustworthy Greebles [*F*(1,127) = 20.76, *p* < .001, η^2^ = 0.14] and a main effect of competence, such that competent Greebles were also rated as more competent than incompetent Greebles [*F*(1,127) = 35.78, *p* < .001, η^2^ = 0.22]. There was no main effect of Trial Type [*F*(1,127) = 2.19, *p* = .141, η^2^ = 0.02]. No significant interactions were observed (all *F*’s < 0.85, all *p*’s > .36).

We again ran paired samples t-tests in order to ensure that when familiar and novel trials were considered independently, significant differences remained in competence ratings. There was a significant difference in competence ratings between competent and incompetent Greebles both when they were familiar to participants (*t*(127) = 5.81, *p* < .001,* d* = 0.51) and when they were novel (*t*(127) = 5.75, *p* < .001,* d* = 0.51). There was also a significant difference in competence ratings between trustworthy and untrustworthy Greebles that were familiar to participants (*t*(127) = 4.52, *p* = .001,* d* = 0.40) and when they were novel (*t*(127) = 3.89, *p* = .001,* d* = 0.34).

## General discussion

We sought to investigate the plausibility of a learning model of first impressions. Whereas some theoretical accounts argue that all first impressions are learned^16,17,27^, others maintain that at least some of the first impressions that we form from appearance are the product of gene-based natural selection^12–15^. Taken together, our studies suggest that learning models have more explanatory power than has thus far been appreciated.

First, we demonstrated that participants can quickly learn to associate the visual features of Greebles with trustworthiness and competence. In our first two experiments, participants were able to learn simple mappings between one type of cue (Family features) and a particular trait (trustworthiness). In Experiment 3, participants also acquired more complex relationships between multiple cues (family and gender features) and multiple traits (trustworthiness and competence). In all experiments, participants generalized their learning about individual Greebles to novel Greebles of similar appearance with little or no decrement. The attribution of character traits to unfamiliar Greebles based on previous appearance-trait experience appears to mirror closely the learning processes involved in spontaneous first impressions from faces.

In our fourth and fifth experiments, we showed that first impressions acquired through learning take on qualities previously assumed to apply specifically to mappings based on innate architecture. That is, participants formed first impressions of greebles in our paradigm even when viewing time was restricted to 100 ms. This accords with recent findings showing that first impressions formed from cultural cues such as glasses occur rapidly^35^. Taken together, these data demonstrate that findings previously thought to support nativist accounts of the origins of first impressions are equally compatible with learning accounts.

Thus, these results provide further support for a learning account of first impressions from faces^16,17,27,35^. The training procedure we used resembles the systematic messages that children receive about the appearance of good guys and bad guys, leaders and follows, jocks and geeks on TV, and in films, comics, and books. In light of our findings, it seems highly likely that this kind of correlated face-trait experience yields equivalent learning about faces, with predictable behavioral consequences. Importantly, this kind of experience would produce similar patterns of first impressions within a culture—consensus impressions.

It has been argued that learning models predict very slow emergence of first impressions over a period of several years^57^. The fact that relatively young children (3- to 4-year-olds) make inferences about the traits of strangers based solely on appearance cues may therefore suggest the presence of innate appearance-trait mappings^37^. However, our results show that appearance-trait mappings can be acquired extremely quickly, and that they readily generalize to novel exemplars. While we cannot rule out the existence of innate mappings between appearance and character judgments, our findings confirm that face-trait mappings can be acquired in the absence of protracted social experience. This view accords with recent accounts emphasizing the importance of cultural learning for cognitive development more generally^58^.

It is striking that participants generalize learning about individual Greebles to novel Greebles of similar appearance with virtually no decrement. Evidently, generalization does not require extensive visual experience with a particular stimulus category; indeed, a lack of perceptual expertise might encourage generalization. Future work may wish to examine how the generalization of appearance-trait rules varies as a function of participants’ expertise with Greebles. This discussion also raises interesting possibilities for future research using faces as stimuli. For example, it would be interesting to investigate whether young children, who have less perceptual expertise with faces than do adults, are more likely to generalize trait profiles to novel individuals based on experience with a few limited exemplars. It would also be interesting to investigate whether adults are more likely to generalize trait profiles from faces with which they have less perceptual expertise, for example those from different ethnicities.

In our paradigm, participants were never told about the different Greeble families and Greeble genders. Thus, it appears that the generalization of learning was down to participants’ ability to detect patterns of similar visual features and extrapolate newly acquired feature-trait rules. However, developmental research makes clear that parents mark some social categories as important in conversation with their children^59,60^. It would be interesting to use the paradigm developed here to test how cultural tendencies to label social groups and mark them as meaningful affects the acquisition of appearance-trait rules.

It is interesting to consider whether similar results would also appear in other cultural contexts. One particularly promising avenue for future research would be to assess whether these results replicate in interdependent cultures. Previous research has suggested that individuals from interdependent cultures rely less on trait inferences than do individuals from independent cultures, and tend to make trait inferences more slowly^61,62^. It is possible, therefore, that the acquisition of first impressions would differ in interdependent cultures.

Our studies suggest that learning models provide a plausible explanation for the origins of all first impressions of character traits from appearance. It has long been known that people attribute character traits to strangers based solely on their appearance. However, the mechanisms responsible are only now becoming apparent. By understanding the learning processes responsible, cognitive science can inform interventions to reduce the influence of these appearance-trait stereotypes.

## Data Availability

The datasets generated and analysed during the current study are available in the OSF repository, https://osf.io/ub6th/?view_only=72831b53995543659182f6db6f254231.
